# Confidence in elections among U.S. local officials: Effects of social trust, partisanship and political ambition

**DOI:** 10.1371/journal.pone.0324794

**Published:** 2025-06-25

**Authors:** Eric Plutzer, Gary Adler, Rebecca Sager, Jonathan S. Coley, Damon Mayrl

**Affiliations:** 1 Department of Political Science, Penn State University, University Park, Pennsylvania, United States of America; 2 Department of Sociology, Penn State University, University Park, Pennsylvania, United States of America; 3 Department of Sociology, Loyola Marymount University, Los Angeles, California, United States of America; 4 Department of Sociology, Oklahoma State University, Stillwater, Oklahoma, United States of America; 5 Department of Sociology, Colby College, Waterville, Maine, United States of America; Hankuk University of Foreign Studies, KOREAREPUBLIC OF

## Abstract

The U.S. public’s confidence in elections is intensively studied in the last decade but little is known about election confidence among locally elected officials, whose roles and community status may influence public opinion. Using a nationally representative survey of local election officials, we compare election confidence among local elected officials with that of the general public. Local elected officials are more likely to trust both local and national elections. We theorize factors that affect local officials’ trust in elections, including partisan context, state leadership election denial levels, and political ambition. We show how social trust, partisan identity, and ambition significantly influence local officials’ confidence that local and national results reflect the intention of voters. We conclude by showing how the relative lack of intensive partisan polarization among local elected officials is especially important at keeping election distrust low among local officials.

## Introduction

Election administration in the United States is highly fragmented, with legal authority deriving mostly from state election laws and implementation delegated to county or other local election officials [1]. By most objective accounts, this system is highly trustworthy. Nearly every voting system has multiple safeguards that ensure continuity of custody of ballots and voting equipment. Increasingly, counties are replacing paperless systems with those that create paper ballots that can be hand counted to confirm electronic totals. The Heritage Foundation [2] tracks cases of voter fraud they believe merit serious investigation and identified a total of 181 “proven” cases over the years 2020–2023 – fewer than one case per year per state (or about 45 per year across more than 3000 jurisdictions). However, as Stewart [3] points out, trustworthy election systems do not automatically translate into trust in election outcomes (see also [4]). Partisanship, media, and electoral results all contribute to a gap between public perceptions and actual electoral performance [3,5,6].

In this paper, we report on election trust among local elected officials. Local elected officials are central players in the politics and policies of American communities [7]. Officials in local communities likely carry significant weight among their constituents and can be consequential for election trust in several ways. First, if large numbers of local officials are election skeptics themselves, they can spread skepticism and may be motivated to ally with political and legal efforts that challenge the legitimacy of elections. Indeed, many electoral integrity legal challenges tracked by Democracy Docket [8] show that petitioners are frequently candidates, incumbents, or former office holders, and a report by the Brennan Center for Justice highlights these “insider threats”—including cases where local elected officials inappropriately accessed election infrastructure or used their knowledge and connections to assist efforts to undermine election confidence [9]. Second and alternatively, if local officials are more confident than ordinary citizens, their official communications can build resilience to election disinformation by tamping down extreme rhetoric, countering false claims, and using their official powers and local social capital to buttress confidence [9–10]. Third, many local officials will seek higher office at the state level where they will be positioned to impact the statutes that govern local election administration, authorize investigations of the conduct of elections, and fulfill other oversight responsibilities [11]. Of course, false confidence – where elections are not trustworthy and officials believe otherwise – would be a serious problem, but this is one that experts suggest is not experienced widely in US national elections [3–6]. For these reasons, it is important to understand local officials’ level of confidence in comparison to that of the public at large and to explore the mechanisms that lead some officials to have more confidence than others.

To do so, we placed two questions on the American Local Leaders Survey, a nationally representative survey of local officials fielded in summer of 2023. In this paper, we first explore whether, compared to the population of citizens, local officials are more confident, less confident, or similarly confident that votes in the 2022 election were counted accurately. We then examine how partisanship, ambition, cues from party elites, and social trust are associated with local office holders’ election confidence.

## Theory and hypotheses

### Officials versus ordinary citizens

Are local elected officials more confident, less confident, or no different from ordinary citizens with respect to the accurate counting of votes cast in the 2022 election? There are two reasons that officials might be more confident than the general public. First, all local officials, no matter their partisan identity, have greater exposure to members of the opposite party in their daily work than ordinary citizens. Interacting with opponents in person, rather than viewing them only as caricatures, can dampen extreme views and motivated reasoning, thereby reducing variance animated by partisanship. Likewise, local officials are also known to discount disagreeable opinions voiced by their constituents [12].

Additionally, if low public confidence is due to misinformation and the exaggeration of the frequency of electoral fraud [13], we might expect local officials to be more confident than the average citizen. This is because of their greater access to reliable information and the greater likelihood that they interact professionally or socially with local colleagues who are directly engaged in election administration. These observations lead us to hypothesize:

H1a: Elected local officials will be more confident in the 2022 election results than ordinary citizens.

Moreover, if the informational mechanism is in play, then this will have its greatest impact among officials in locales with primarily responsibility for election administration. This will mostly affect county officials, as counties have the primary responsibility for election administration in most US states. But there is considerable variation with many cities having primary responsibility, particularly in New England and in some larger cities such as Chicago, Milwaukee, and others.

H1b: Officials in locales that administer elections will have greater confidence in election outcomes than other officials.

On the other hand, most local officials are unpaid or part-time public servants, making them similar to engaged citizens they represent. From this perspective, public officials would have beliefs that are quite like those of ordinary citizens [14], and we might expect that we cannot reject the null hypothesis of no effect.

H1_0_: Elected local officials will be no more confident in the 2022 election results than ordinary citizens.

### Partisan effects on levels of confidence

Partisanship is a powerful predictor of virtually all political beliefs and attitudes in the contemporary United States. We theorize three mechanisms through which partisanship can impact officials’ confidence in elections.

### Individual partisanship effects among officials

Recent surveys of the voting eligible public show that Republican citizens show lower confidence in elections than Democrats [15,16]. Republican officials may genuinely believe that fraud, technology, or incompetence results in substantial errors in the counting of votes, extrapolating from documented inefficiencies in the maintenance of voter rolls, and use of electronic machines that cannot produce a written record. [10]. Such legitimate worries were manipulated by President Trump in 2020, and amplified by other GOP officials, with numerous false claims about voter fraud introduced as well. If elected officials tend to have stronger partisan ties than the average citizen, then this should lead to substantial polarization as reflected by large associations between partisan identification and election confidence beliefs.

H2a: Local officials who identify as Republican have lower electoral confidence than local officials who identify as Democrats.

Alternatively, it is possible that due to differential sources of information, Democrats and Republicans sincerely perceive different realities with respect to the accuracy of tabulated votes in their communities and in the nation as a whole (e.g., [17]). This would also lead to hypothesis H2a.

### Partisan contextual effects

In addition to individual partisan identification, partisanship may also operate through contextual mechanisms. Local elected officials are not only drawn from the local environment but also seek to send signals that they support their party’s prevailing view. The pressure to do so will be strongest in areas where one party is clearly dominant [18]. We therefore expect officials working in local contexts that voted heavily for Joe Biden in the 2020 election will express less election skepticism than officials in areas where Donald Trump did especially well. This contextual effect could operate above and beyond personal party identity.

H2b: As the share of the local vote won by Donald Trump increases, local officials’ electoral confidence will decline.

Contextual effects may be amplified for the subset of local officials who are open to or actively considering running for higher office. Political ambition increases officials’ attentiveness to local opinions generally (e.g., [19]), and they may be especially attentive to their party’s base. We expect politically ambitious Republican local officials to display their party’s election trust stance with greater intensity in areas of Republican voting strength.

H2c: As the share of the local vote won by Donald Trump increases, local officials’ electoral confidence will decline, with the effect being stronger among ambitious officials than non-ambitious officials.

### Social trust

Confidence and trust are closely related concepts (e.g., [20–21]). High confidence in institutional performance reflects *trust* in the capability and impartiality of individuals running those institutions. Within most nations, individuals who are high in generalized social trust (e.g., think that most people can be trusted) have higher confidence in political institutions [21] and the people running those institutions [22–23]. In that light, confidence that votes were counted accurately and that election outcomes reflect the intentions of voters could be largely determined by, or simply a reflection of, social and institutional trust (see [24–26]). In this case,it is useful to see how strongly social trust predicts election confidence and it is important to account for social trust as a potential confounder. The survey we utilize has a good measure of social trust to explore this argument, and we therefore hypothesize:

H3: As generalized social trust increases, confidence in elections increases.

#### Elite cue effects.

An additional mechanism suggests that partisanship can operate when local officials take cues from the actions and rhetoric of party elites in the party power structures in their states. In the case of the accuracy of the 2020 presidential election, we know of no high-level Democrats who claimed there was widespread voter fraud. However, GOP elites were divided, and we can take advantage of that within-party variance to explore the effect of elite cues. Specifically, many Republican candidates for statewide office (Governor, Secretary of State and US Senator) and for the US House of Representatives took public positions on electoral integrity during the 2022 campaign [27]. Public pronouncements by elites could influence local officials in one of two ways, either to express skepticism (if elites were election deniers) or to support the legitimacy of the vote (if elites conceded that Joe Biden was legitimately elected President). That leads us to hypothesize:

H3a: Among Republican officials, as election denial among state elites increases, confidence in elections will decrease.

Moreover, this effect might be especially strong among *ambitious* Republican local officials. Those who aspire to higher office often have one eye on their local constituents and another on the political environment of the state or nation [28–29]. They might strategically consider the benefits of adopting positions that will earn them the support of partisan colleagues to earn nominations for federal and statewide office.

H3b: The effect of state official denialism is stronger for those expressing interest in pursuing higher elected office than among the non-ambitious.

## Data and methods

### Study description

Our data come from a larger study, which fielded a national probability sample survey of elected and appointed municipal, county, and school district officials. This paper relies only on the subsample of elected officials – members of municipal and county councils, mayors, county executives (if elected), and a small number of other elected officials (mostly county sheriffs). The sample intentionally does not include local officials whose sole or primary responsibility is election administration (e.g., “Director of Elections,” “Elector Board,” “Registrar”). The sample is based on a two-stage process where we first selected a probability sample of 801 cities and 616 counties, where the largest places were selected with certainty, all but the smallest places were selected with probability proportional to size (PPS), and tiny places selected with a PPS oversample. The full details on the sampling design are provided in Appendix A in S1 Appendix. In a second stage, all officials in each selected locale with a relevant job title were sampled. This research was deemed exempt by the IRB of Penn State University (IRB study number 00020506).

Recruitment was based on the tailored design method [30]. For the 95% of officials for whom we had a valid email, we sent a pre-notification letter sent by post, an email invitation to participate, three email reminders, and a “study is closing soon” final email. The 5% lacking email received a pre-notification letter with a link to our web survey, a reminder postcard, and a “study is closing soon” postcard. Pre-notification letters were mailed July 3, 2023 and surveys were completed between July 5 and September 19, 2023 (97% of surveys were completed before August 16, but the survey remained open for very late returns). The first screen of the survey explained that the survey was voluntary, any question could be skipped and was anonymous. Respondents affirmed they were over 18 years old and currently serving as a local official by giving implicit consent (advancing to the next screen and first question). A more detailed description of the fieldwork and informed consent is contained in Appendix B in S1 Appendix. Officials completed a survey hosted by the corresponding author’s home institution Qualtrics platform. The median official completed the survey in 34 minutes (including any time away from the survey).

The overall response rate (AAPOR RR4) for city and county officials was 9.9%. As detailed in Appendix C in S1 Appendix, response rates were higher for officials with a valid email, and for officials in communities with higher levels of education. However, there were no systematic patterns of non-response based on community race, ethnicity, religion, or county level vote in the 2020 election (see S1 Appendix Tables C1 and C2).

The sample was designed to be self-representing with cities selected in proportion to the number of constituents (that is, the cities with a population of 100,000 had a probability of selection exactly double that of cities with a population of 50,000). Ignoring non-response, results generalize to constituents, not officials. Based on the propensity model reported in table C1 in S1 Appendix, we calculated a non-response adjustment weight, and all analyses in this paper are weighted.

### Dependent variables

Our key dependent variables are answers to two questions that measure confidence in the election. These questions are the second and fourth items in the question set used in the MIT/Caltech Survey of the Performance of American Elections [31–32], a longstanding national survey of electoral performance from the citizen’s perspective. Our first item asks, “Thinking about last year’s mid-term elections in November, how confident are you that votes in your **county or city** were counted as voters intended?” Of the elected officials who completed our survey, 83% said they were “very confident” (see Table 2 for more details). Our second item similarly asks, “Now think about vote counting throughout the **country**. How confident are you that votes nationwide were counted as voters intended?” As shown in Table 2, only 54% said they were very confident and fully 19% said they were “not too confident” or “not confident at all.” The full question wording for this and all other survey questions used in this paper can be found in Appendix E in S1 Appendix.

In samples of the general public, public confidence as measured by these questions consistently shows greater confidence in local elections than national elections [32]. Moreover, answers to both items are sensitive to election outcomes, with citizens identifying with the losing party showing less confidence than when their candidate prevails [3,32]. By contrast, smaller shifts in these questions are associated with actual administrative performance (e.g., lower confidence expressed by residents of states that report the vote late) [33]. Our interest lies in understanding variation among officials regarding confidence in a specific election cycle, especially factors that operate after controlling for partisan identification.

### Key independent variables

#### Level of local government.

We use a binary variable to indicate whether the official is part of county (1) or municipal (0) government. The level of government was identified in the position listings of the sampling frame (See Appendix A in S1 Appendix for details).

#### Partisan identification.

We utilize the seven-point party ID measure used in the American National Election Study and the General Social Survey, where 0 indicates strong Democrats and 6 indicates strong Republicans. Because partisanship is central to many of our core hypotheses, we show the sample distribution in Table 1. Notably, “strong” partisans represent a majority (56%), but with considerable variation in intensity.

**Table 1:** Partisan identification among local elected officials.

*A. Nominal identification*		*B. Strength of identification*	
	Percent		Percent
Strong Democrat	31.8	Strong partisan	56.2
Not strong Democrat	11.1	Not strong partisan	24.1
Lean to Democrats	6.7	Lean towards one party	14.9
Independent	4.8	Independent or other	4.8
Lean to Republicans	8.2	Total	100.0
Not strong Republican	13.1		
Strong Republican	24.5		
Total	100.0		

#### Social trust.

**Fig 1:**
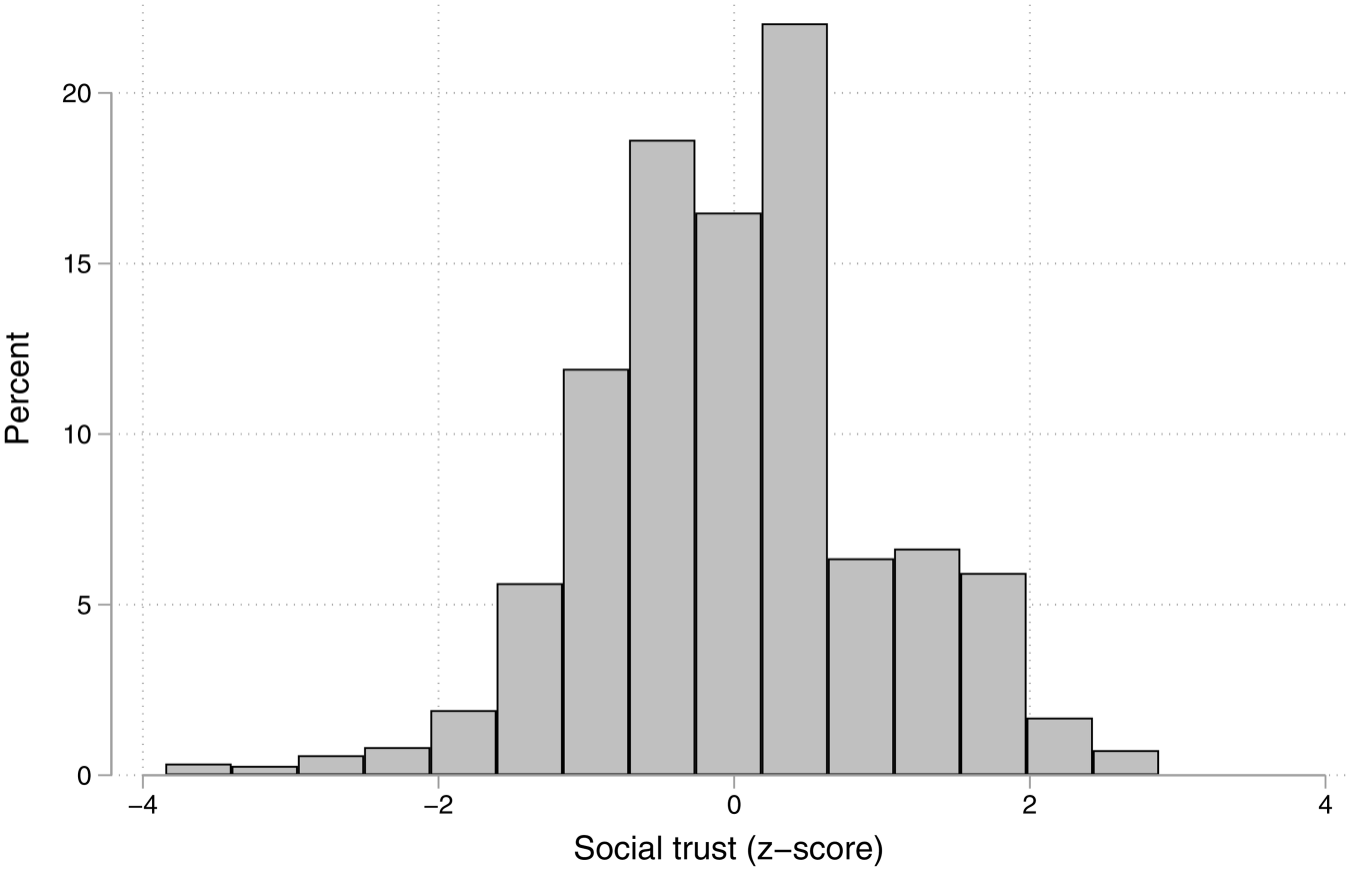
Distribution of social trust scale (N = 743).

Social trust is measured with five questions we adapted from those used in the World Values Survey. Officials were asked to say how much they trust their family, neighbors, strangers, people from a different religion, and people from a different political party. For each, they could indicate they trusted “completely,” “somewhat,” “not very much,” or “not at all.” Exploratory factor analysis suggested that the items are well represented by a single latent factor and the resulting scale (based on factor scores using the Bartlett method) has an Omega reliability of 0.61 (Cronbach’s alpha = 0.60). The scale is standardized, with a mean of zero and a standard deviation of one (see Fig 1). Those with maximum scores display high trust in both close and distal contacts, those in the middle tended to show high trust in neighbors and family, but intermediate levels of trust in strangers and those from a different political party. The small number of very low scores are officials who distrust all groups, including their family and neighbors.

#### Political ambition.

Political ambition is a binary variable coded 1 if the official indicated that they are actively considering or open to the possibility of seeking higher elected office than the position they held at the time of the interview and coded 0 for those expressing “no interest.”

#### Partisan context.

For both municipal and county officials, we measured the Republican share of their county’s 2020 presidential two-party vote. Anticipating our regression models, we recoded this into increments of 10 percentage points (e.g., 53% of the county vote is coded as 5.3 and 63% as 6.3); in this way, a regression slope will connote the change in electoral confidence associated with a ten-percentage point increase in Republican share of the vote.

#### State leadership election denial score.

To capture elite cues around election denial, we utilize data compiled by journalists at FiveThirtyEight [34]. They produced a publicly available dataset on the population of every Republican candidate for Senate, House, governor, attorney general and secretary of state running in the 2022 general election. They reached out to each candidate and used their answers in combination with “news reports, debate footage, campaign materials and social media” to classify candidates into six categories ranging from those who “explicitly said the 2020 election was illegitimate and/or took legal measures to try and overturn the election,” to those who “accepted Biden’s victory without reservations.” We converted these to an ordinal score ranging from 0 (fully accepted) to 3 (fully denied) and then calculated the mean candidate score for each state, which we take as a summary of the public stances of Republican party leadership. The scores run from 0.67 (Nebraska) to 3.00 (West Virginia) with an overall mean of 1.86 and a standard deviation of 0.45.

### Control variables

Our multivariable analyses include a number of covariates that might confound the relationships among election confidence, partisanship, and trust. These include age, whether the official is married or in a civil union; whether they have children under 18 living in their household; whether they or someone in their household ever served in the U.S. military, reserves, or National Guard; highest level of school completed; whether the official identifies as Hispanic; a measure of community size grouped into quartiles; an ordinal measure of the official’s number of years in their current office; and whether the official is the highest elected official in their jurisdiction (e.g., Mayor, coded 1). (We use a measure of Hispanic for race/ethnicity because most non-white racial identities have low numbers of cases and, due to close overlap, African Americans are largely captured by the RELTRAD religious tradition classification of Black Protestant instead).

We also control for religiosity and religious tradition. Religiosity is a composite scale variable constructed from frequency of personal prayer other than at meals (daily, weekly, monthly, a few times a year, never), frequency of religious service attendance other than at weddings and funerals (every week, almost every week, once or twice a month, a few times a year, never), the extent that religion provides guidance in day-to-day life (none, some, quite a bit, a great deal), and extent they are a spiritual person (not at all, slightly, moderately, very). Each measure was converted to a z-score (M = 0, SD = 1) and then averaged. Religious affiliation is measured using a variation of the RELTRAD classification [35], with seven categories (Conservative Protestant, Mainline Protestant, Black Protestant, Catholic, Jewish, Other Religion, No affiliation). Note that the RELTRAD category of Black Protestant churches is nearly collinear with Black racial identity, and we cannot estimate models with both; as a result, we do not include a dummy variable for identifying as Black. Finally, gender and sexual identity are based on two questions. The first is “Are you a: Man, Woman, Something else,” and the second asks “Which of the following best represents how you think of yourself? Lesbian or gay, Heterosexual or straight, Bisexual, Prefer not to answer, [or] Don’t know.” Operationally we created a dummy variable for female identification, and a dummy variable coded 1 for those selecting Lesbian or gay or Bisexual on the sexual preference measure or selecting “something else” on the gender identity measure. (We are mindful that this operationalization lumps together heterogeneous sexual minority statuses. In a general population survey of similar size, best practices would make finer distinctions. However, this sample skews older than the general population and we have limited subsamples: n = 9 individuals who reported their sex as something other than male or female (2 of whom also identify as lesbian or gay and 1 who identifies as bisexual); 24 men and 10 women who identify as lesbian or gay; 7 men and 10 women who identify as bisexual. Because this paper employs these measures as control variables, we felt collapsing disparate identities into a single LGBQT indicator that encompasses answers of “something else” on the sex question was the best approach.)

## Results

### Officials compared to registered voters

To address our first research question, we pooled data from our own survey of elected officials with comparable data from the November 2022 Survey of the Performance of American Elections and its sample of registered voters.

Comparing columns 1 and 2 of Table 2, we can see that 83% of elected officials were very confident that votes in their community were counted accurately. Only about 3.5% of elected officials expressed that they were “not at all confident” or “not too confident.” In contrast, more than three times as many – 11% – of registered voters expressed low confidence and only six in ten were very confident. Moving to columns 3 and 4, we see that both populations show lower confidence that votes in the nation as a whole were counted accurately than votes in their community. Only 37% of registered voters were very confident, substantially fewer than the 54% of local elected officials. We conducted a formal test by pooling the two data sets and estimating ordinal logistic regressions for each confidence question, with a source indicator taking a value of 0 for the elected official sample and 1 for the MIT/Caltech public sample. For both dependent variables the odds ratio was statistically significant and negative, indicating lower confidence among the public (for national confidence, OR =.597, t = − 6.10, p < .000; for local vote confidence OR =.314, t = − 10.34, p < .000). These results were similar when we also added state fixed effects. Thus, these results support hypothesis 1a.

**Table 2:** Confidence in local elections (columns 1 & 2) and confidence in national election (columns 3 & 4) for samples of local elected officials and sample of registered voters (column percentages).

	Votes in your county or city were counted as voters intended		Votes nationwide were counted as voters intended	
	Local elected officials survey	Registered voters survey	Local elected officials survey	Registered voters survey
Not at all confident	1.3	4.4	8.5	13.2
Not too confident	2.2	6.7	10.8	17.4
Somewhat confident	13.2	29.2	26.4	32.1
Very confident	83.3	59.7	54.2	37.3
Total	100.0	100.0	100.0	100.0
Obs	1,170	9,784	1,161	9,771
Pearson Chi-sq(3 df) = 246.9359			Pearson Chi-sq(3 df) = 129.3840	

Note: Source for columns 1 & 3 is the authors’ 2023 survey of elected officials; source for columns 2 & 4 is the November 2022 Caltech/MIT Elections Lab’s Survey of the Performance of American Elections. Column percentages are weighted.

This exploration tells us two things. First, both groups are more confident that votes in their own communities were counted accurately than they are that votes elsewhere were. Second, elected officials are much more confident in elections than the voters they represent. (This pattern is not due to different geographical representation; in results reported in the appendix, we show that the same pattern results after we account for state fixed effects – indeed, state fixed effects have virtually no impact on the gap between officials and voters).

### What drives confidence in local and national elections?

We now move on to test our hypotheses concerning mechanisms of partisanship as well as trust. Our empirical strategy relies on regressing each of our confidence measures on the array of key independent and control variables. Because each outcome is ordinal, we use ordinal logistic regression with survey corrected standard errors (using Stata’s Taylor linearization estimate of standard errors to account for weighting). When estimating the model for confidence in national elections, we include confidence in local elections as a control so that we can isolate effects on national confidence net of local confidence.

**Fig 2:**
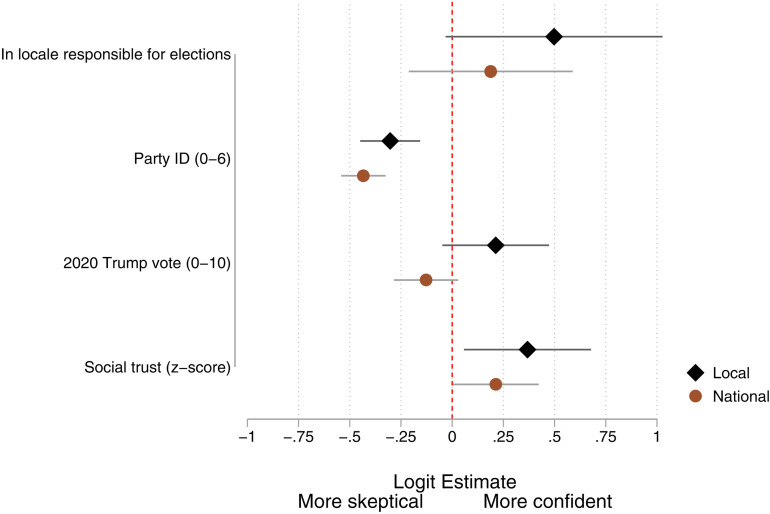
Ordinal logistic regression of confidence in elections.

The results for the entire sample are summarized in Fig 2, where the dots represent the odds ratio associated with a one-unit change in each independent variable. Estimates for control variables are omitted from the graph and are reported fully in Table G.2 in S1 Appendix.

#### Are officials more confident if serving a jurisdiction responsible for elections?.

Hypothesis 1b suggests that officials who are more likely to have primary or secondary connections to local election administrators would be less susceptible to claims of election fraud or administrative incompetence. This hypothesis is partially supported. The top black diamond in Fig 2 shows that such officials are more confident that local votes were counted accurately, but the effect falls short of conventional levels of significance (*p* = 0.065). However, this additional boost does not translate into increased confidence in national elections.

#### How is partisanship associated with confidence in elections?.

The next variables are individual partisan identification (H2a) and the local partisan context (H2b, operationalized as Donald Trump’s 2020 share of the county vote). Clearly, individual partisanship matters, with each one-unit shift from 0 (strong Democrat) to 6 (strong Republican) resulting in a significant decline in confidence in both local and national election results. Holding all else at the mean, a strong Republican has a 0.84 probability of being very confident in local elections in comparison with a typical strong Democrat whose probability of being very confident is 0.97. The effects of partisanship on national election confidence are substantially larger. Holding all else at the mean, a typical strong Republican has a 0.20 probability of being very confident in the national election count in comparison with a typical strong Democrat whose probability of being very confident is 0.78.

In contrast to individual partisanship, the prevailing partisan environment has weak and inconsistent effects and the net impact of Trump’s share of the vote is not significantly different from zero, providing no support for H2b.

Our third hypothesis concerned social trust, and this is supported. Elected officials who are high in social trust are significantly more likely to have confidence in local (*p* = 0.02) and national (*p* = 0.047) election results. Consider two officials who are otherwise identical, but one scores a standard deviation below the mean on trust and the other a standard deviation above. The former has a 0.90 probability of being very confident in their local elections and the latter a probability of 0.95. When expressed in odds, the effects are slightly smaller when it comes to national elections, but because the overall odds are roughly even, the substantive effects are considerable. Officials with trust scores at 1 SD below and 1 SD above the mean have probabilities of high confidence being 0.45 and 0.56, respectively.

#### How does party elite denialism and political ambition impact Republican local office holders?.

**Fig 3:**
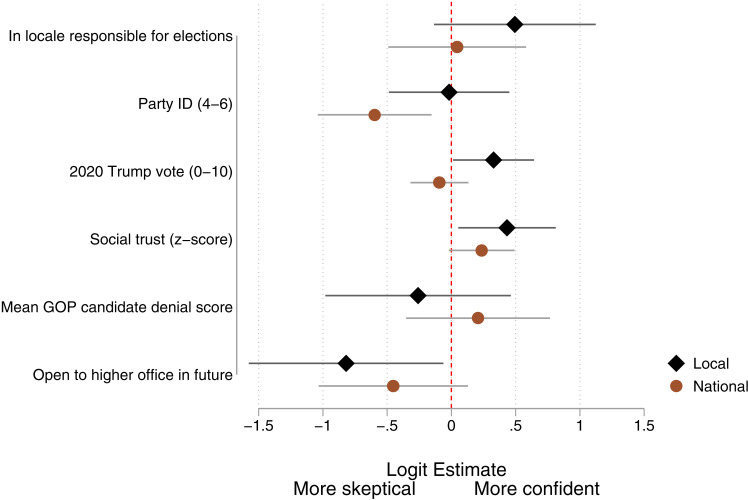
Ordinal logistic regression of confidence in elections (Republicans only).

In this section we focus on the subsample of Republican local elected officials. In contrast to the Democratic party, Donald Trump and other GOP elites have consistently sought to call attention to alleged electoral fraud and the “rigging” of election procedures – efforts that pre-date the events of January 6, 2001 by more than four years. Nearly a month before the 2016 election, Trump tweeted “The election is absolutely being rigged” [36]. Shortly after winning that election, he signed an executive order creating a Presidential Advisory Commission on Election Integrity. For this reason, two of our hypotheses concerning partisanship focused on GOP officials. We thus re-estimated our model for the subsample of Republicans (including leaners). The key estimates are summarized in Fig 3 (full results, including for controls, are reported in Table G3 in S1 Appendix).

We first note that the smaller sample (N = 256) results in greater uncertainty in the estimates. We also note that, compared to the full sample analyses, the effects of local responsibility and social trust are similar, the effect of individual partisanship is now diminished for confidence in local elections, but with a compensating increase in the effect of local Trump support.

We now turn to the hypotheses that are specific to Republicans. H4a follows from the substantial cross-state variation in election denial rhetoric and proposes that Republican office holders would take cues from party elites in their states. The plot shows that variation in party elite position had no impact on officials’ confidence in either local or national elections, so H4a is rejected.

Hypothesis H4b predicted an interaction between state elite denialism and ambition, reasoning that ambitious officials would have their eye on cues from elites who are serve as gatekeepers for higher office. But we found that this interaction was not statistically significant in predicting either local or national election confidence (*p* > 0.50 in both instances, as shown in Table G4 in S1 Appendix).

However, Fig 3 shows a main effect of ambition with ambitious GOP local office holders being less confident in national elections than their less ambitious counterparts. Indeed, the estimated probability that an ambitious GOP official expresses high confidence in the national election results is just 0.12.

## Discussion

Public confidence in elections is one of the foundational pillars sustaining citizen support for democracy. Summarizing the literature, McAllister and White write, “A lack of integrity in electoral institutions undermines the basic principles of fairness and impartiality that lie at the heart of a well-ordered and functioning democratic society. Moreover, a political culture that sustains widespread corruption harms democratic citizenship, by weakening democratic knowledge and citizens’ sense of efficacy and trust in the institutions of government” [37, p. 79; 38].

Previously, much of the scholarly literature about electoral trust focused on emerging democracies throughout the world. However, popular confidence in US elections has become the focus of intensive research and policy debate. Our analysis focuses on a potentially influential set of actors who can impact the views of their constituents: elected officials of local governments.

### Limitations

To our knowledge, our survey is the first national probability survey of local officials to explore levels of election confidence. That said, our cross-sectional research design has several implicit limitations.

First, our officials were interviewed eight months after the November 2022 election. The opinions of respondents to the MIT/Caltech survey might have shifted in those eight months so the comparison between the two surveys is not perfect. However, a major shift in the general public’s election confidence in eight months seems very unlikely. In the period from 2012 to 2022, the gap between the highest and lowest levels of this variable is only 15 percentage points (23% in 2014 versus the high point of 38% in 2020. Thus, we think that only a small portion of the 23 point gap between officials and the public could conceivably be explained by the eight-month span between the surveys.

Second, we cannot account for the possible role of election trust in citizens’ selection into the pool of public officials that we sampled. It is possible that those with high election confidence are more likely to seek public roles or be more likely to respond to our survey. On the latter, the analyses reflect weights that account for the propensity to respond to the survey and hopefully minimize this potential self-selection bias. It is possible that election winners naturally express confidence in election outcomes as a consequence of motivated reasoning.

Third, it is possible that confidence in elections is a stable trait learned earlier in life whose changes induce corresponding changes in social trust. If this were the case –that is, if election confidence were similar to confidence in parliament and other institutions [22–23] then our treatment of social trust as an independent variable would be unjustified.

### Implications

We found that local office holders are substantially *more confident* in local and national election results than the general public. We suspect that the interactional nature of local government work, which occurs across partisan lines, along with greater access to information, both shape local officials’ relatively high amounts of election trust. Consistent with this, officials’ jurisdiction level predicted election trust in a straightforward way: officials in governments that administer elections were more confident than others. We attribute this to the familiarity that these officials are likely to have with election staff and operations in their city or county, even though our sample members are not directly involved. However, this knowledge does not appear to insulate officials from other sources of distrust and polarization that significantly impact confidence in election results outside of one’s own community.

A second important factor is social trust. Notably, our measure of social trust was completely uncorrelated with partisanship (r = 0.01, ns) and it showed that those who are wary of others – strangers, those of a different religion or political affiliation – are especially likely to doubt the results of the 2022 election. Social trust, at least for this outcome, extends its reach to confidence in institutions, even in a sample that is well educated and has access to information from their professional contacts and associations. Local elected officials could act as models of trust for local communities. Unfortunately, social trust has steadily declined since the early 1970s (e.g., [39]), and it is unlikely that local officials are immune to this long-term trend.

However, as with the general public, the most powerful predictor of election confidence was partisanship, with Republican local officials less likely to express confidence both local and national election results. Even in the context of a non-partisan, confidential survey, the large majority of officials followed the party line. However, our hypothesis that partisanship could influence officials through cueing by state party elites was not supported. Notably, differences in strength of partisan identity were also significant in the Republican sub-sample analyses, but only regarding national elections, suggesting the continued salience of commitment to the “big lie” within the party as a marker of Republican identity. This interpretation is supported by one other finding—that political ambition is negatively correlated with trust in national elections, but only among Republic officials.

Two conclusions may seem contradictory at first glance. We showed that Republican partisans have extremely low confidence that ballots were counted accurately outside of their own jurisdiction, but also that election confidence is higher among elected officials than ordinary citizens. These conclusions can both be true because, in July 2023, most Republican identifiers were weak identifiers or leaners who opted for a political label only after probing. By comparison, a similar survey of members of Congress would reveal only a single-digit presence of independents and weak identifiers (e.g., Senators Manchin and Sinema before declaring themselves Independents). The distribution of party identification simply appears different among local elected officials than among elected officials in higher levels of government, with a larger share of local officials displaying weaker partisan ties. Thus, local Republican officials may buffer the more extreme election denial rhetoric of their national leaders.

But there also is a less optimistic reading to this result. If moderates are increasingly forced out of their parties, discouraged from running for local office, or compelled to toe the party line to fulfill their ambitions, the moderate middle could become hollowed out. If that occurs, local officials will become increasingly polarized. Given the current positions of party leaders, this would result in election skeptics replacing confident officials over time.

## Supporting information

S1 Appendix This file contains Appendix A-F and Table G.1-G.4. (DOCX)
