# Supplementary material for: Confidence in elections among U.S. local officials: Effects of social trust, partisanship and political ambition
Source: PLoS One. 2025 Jun 25;20(6):e0324794. doi: 10.1371/journal.pone.0324794 (PMC12194158; doi:10.1371/journal.pone.0324794)
Supplement: S1 Appendix — (DOCX) [file pone.0324794.s001.docx]

**Supporting Information for:**

**Confidence in Elections Among U.S. Local Officials:**

**Effects of Social Trust, Partisanship and Political Ambition**

Appendix A: Sampling

Appendix B: Survey Recruitment

Appendix C. Non-response Analysis

Appendix D. Analysis Weights

Appendix E. Exact Question Wording

Appendix F. Non-survey Constructed Variables

Appendix G. Supplementary Tables

**Table G.1. Descriptive statistics**

**Table G.2 Full models used to produce figure 2** (logit estimates with standard errors in parentheses).

**Table G.3 Full models used to produce figure 3** (logit estimates with standard errors in parentheses).

**Table G.4 Models used to test ambition × denialism interaction** (logit estimates with standard errors in parentheses).

**Appendix A: Sampling**

This paper reports data from the larger survey that included samples of appointed officials and school superintendents. The key election confidence questions were only asked of elected officials, so this appendix describes the sampling and fieldwork relevant to the elected officials subsample.

County and Municipal Jurisdictions

We began by identifying populations of local governments and their officials:

1. Municipalities. This term encompasses incorporated areas, minor civil subdivisions, and consolidated city/county governments (colloquially referred to as cities, towns, villages, etc.).

Elected officials included:

- Mayors (or the highest-ranking elected official)
- Elected members of the city’s governing body (council members)

2. Counties. This includes US counties and county-equivalents, such as Louisiana parishes.

Officials include:

- Elected members of the city’s governing body (commissioners, members)
- Mayors (or the highest-ranking elected official)
- Elected heads of law enforcement (sheriffs)

In each instance, we utilized a two-stage sampling design, whereby local governments are selected in the first stage, and *all* eligible officials in each selected local government are recruited for survey participation. This design was adopted to open the possibility of analyzing within-community heterogeneity – how much variation and disagreement do we see *within* communities in addition to examining variation *across* communities.

**A.2. First stage sampling of local governments**

***A2.1. Municipality sampling***

To select cities, we began with a US Census Bureau list of 16,789 municipalities (incorporated places, minor civil subdivisions and eight consolidated city/county entities) that were present in our sampling frame. See [1].

Our first stage sampling proceeded as follows:

Consistent with the PPS approach, we classified all cities into one of three sampling strata:

1. *Municipalities sampled with certainty*. These are the N=101 municipalities with populations of 225,000 or greater.

Each of these has a probability of selection (*f*) for any municipality j is equal to exactly 1:

2. *Municipalities subject to probability sampling*. These are N=10,504 municipalities with populations of 2,000 to 225,000. We utilized Stata’s **gsample** procedure to select a probability sample of n=700 with selection probabilities proportional to size.

Each of these has a probability of selection (*f*) for any municipality ***j*** equal to:

$$f_{j}=\frac{{pop}_{j}}{\sum{pop}_{j}}\times n$$

Or approximately:

$$f_{j}=\frac{{pop}_{j}}{178,288,917}\times700$$

3. *Small municipalities* (N=6,184) with populations of under 2,000 residents. We drew an oversample of n=70 from this frame, also with probability proportional to size.

Each of these has a probability of selection (*f*) for any city ***j*** equal to:

$$f_{j}=\frac{{pop}_{j}}{\sum{pop}_{j}}\times n$$

Or approximately:

$$f_{j}=\frac{{pop}_{j}}{8,401,668}\times70$$

If we wish to generalize to the government characteristics experienced by the *population of citizens*, the sample is approximately self-weighting.

The resulting sample (n=863 cities) has the following characteristics:

They represent the full range of very small, small, medium, and large cities. Quartiles are bounded by the following population thresholds:

Quartile 1. (Very small) From 728 to 11,229

Quartile 2. (Small) From 11,229 to 36,684

Quartile 3. (Medium) From 36,684 to 109,705

Quartile 4. (Large) Above 109,705

To provide some intuition, municipalities with 10,000 individuals had approximately three chances in one hundred to be selected, while a municipality with 100,000 residents had about three chances in ten. A municipality with just under 225,000 individuals had two chances in three.

***A2.2. County sampling***

To assist in our two-stage sample of local county officials, we contracted with PowerAlmanac, a direct mail and research firm that maintains a large, accurate, and current database of municipal and county officials.

At the time we accessed the database (April 3^rd^, 2023) Power Almanac had listings for 26,670 officials in 3,003 counties. The list is updated monthly and 72% of the entries had been updated or verified in the 101 days prior to our download, with 99% within 180 days.

Our first stage sampling proceeded as follows:

We classified all counties into one of three sampling strata:

1. *Counties sampled with certainty*. These are the N=179 counties with populations of 350,000 or greater.

Each of these has a probability of selection (*f*) for any city j is equal to exactly 1:

2. *Counties subject to probability sampling*. These are N=2,163 counties with populations of 10,000 to 350,000. We utilized Stata’s **gsample** procedure to select a probability sample of n=360 with selection probabilities proportional to size.

Each of these has a probability of selection (*f*) for any county ***j*** is equal to:

$$f_{j}=\frac{{pop}_{j}}{\sum{pop}_{j}}\times n$$

Or approximately:

$$f_{j}=\frac{{pop}_{j}}{128,727,509}\times360$$

3. *Small* counties (N=664) with populations of under 10,000 residents. We drew an oversample of n=80 from this frame, also with probability proportional to size.

Each of these has a probability of selection (*f*) for any county ***j*** is equal to:

$$f_{j}=\frac{{pop}_{j}}{\sum{pop}_{j}}\times n$$

Or approximately:

$$f_{j}=\frac{{pop}_{j}}{3,740,074}\times80$$

If we wish to generalize to the government characteristics experienced by the *population of citizens*, the sample is approximately self-weighting.

The resulting sample (n=616 counties) has the following characteristics:

They represent the full range of very small, small, medium, and large cities. Quartiles are bounded by the following population thresholds:

Quartile 1. (Very small) From 1,628 to 39,220

Quartile 2. (Small) From 150,697 to 150,697

Quartile 3. (Medium) From 150,697 to 386,694

Quartile 4. (Large) Above 386,694

**A.3. Identification of eligible officials from each selected government**

Within each sampled government unit, we relied on a list provided by PowerAlmanac, a direct mail and research firm that maintains a large, accurate, and current database of municipal and county officials.

At the time we accessed the database (April 3^rd^, 2023) Power Almanac had listings for 137,212 officials. The list is updated monthly and 59% of the entries had been updated or verified in the 100 days prior to our download, with 99% within 180 days.

**APPENDIX B: Survey Recruitment**

**B.1. Schedule of email and postal contacts**

Once our sample was selected, we pursued a multiple contact strategy to invite sampled officials to participate and to follow up with multiple reminders.

Our sample file contained email addresses for approximately 95% of sampled officials. Those officials received a pre-notification letter, reminder postcard, and five email contacts. The 5% of officials for whom we lacked an email received the pre-notification letter, the reminder postcard, and an additional reminder postcard.

|  | Officials with email | Officials without email |
| --- | --- | --- |
| Pre-notification letter | July 03, 2023 | July 03, 2023 |
| Email invitation | July 11, 2023 |  |
| Email reminder #1 | July 13, 2023 |  |
| Postcard reminder #1 | July 14, 2023 | July 14, 2023 |
| Email reminder #2 | July 15, 2023 |  |
| Email reminder #3 | July 19, 2023 |  |
| Final “study is closing” email | July 28, 2023 |  |
| “Study is closing” postcard |  | July 31, 2023 |
| Last questionnaire completed | September 19, 2023 | August 15, 2023 |

**B.2. Recruitment documents distributed by post:**

Prenotification

Reminder postcard #1

Reminder postcard #2 (only non-email)

**B.3. Recruitment messages distributed by email (subject lines):**

Welcome to the Local Leaders Study

Please share your views on local governance: An invitation to the American Local Leaders Study

We want to hear from you for the Local Leaders Study

Your experiences as a local leader matter. Please participate in our study.

Our study is closing soon. Please complete the Survey of American Local Leaders

**B.4. Consent (first) screen of survey:**

**Welcome to the American Local Leaders Survey!**

You can take this survey on any device. A large screen and keyboard will help you take the survey more quickly. At the end, you’ll have a chance to request a copy of the study results.

Your participation in this research is voluntary. You may decide to stop at any time and you do not have to answer any questions that you do not want to answer.

Your participation is anonymous, and your individual responses will be kept strictly confidential. 

By taking this survey you are affirming that you are 18 years old or older and that you currently are a local government official. 

**Question or Concerns?**
If you have questions or concerns, please contact the principal investigator for this study, Gary Adler, Ph.D. He is in the Department of Sociology and Criminology at Pennsylvania State University, University Park, PA.

You can reach him at nationalsurvey@psu.edu

**Appendix C. Non-response Analysis**

**C.1. Calculating response rates**

**C.1.1. Dispositions**

Criteria and estimates are based on AAPOR [2] criteria for email Surveys of Lists of Specifically Named Persons. We calculated counts for the following dispositions (the subscript ***mc*** identifies the subsample of municipal and county officials):

- **Completed** interview (N_all_ = 1,196) [N_mc_ = 1,096]
  - Respondent completed 90% of the survey (Qualtrics progress >= 90)
- **Usable** partial interview (N_all_ =142) [N_mc_ = 127]
  - Completed 35% of the survey (Qualtrics progress >=35 & <90)
- **Refusals** (N_all_ =245) [N_mc_ = 228]
  - Immediate breakoffs (progress < 5%, N=45) [N_cc_ = 42]
  - Early breakoffs (progress >5% & < 35, N=200) [186]
- **Nonrespondents** of unknown eligibility
  - Nonrespondents presumed as eligible (N_all_ =15,701) [N_mc_ = 12,094]
- **Ineligible** no interview (disposition 3.0). [N_mc_ = 961]
  - Hard bounce on all five email distributions (N_all_ =923) [N_mc_ = 629]
  - Non-respondent with returned postal letter (N_all_ =351) [N_mc_ = 341]

**C.1.2. Estimating the proportion of non-respondents who were eligible to participate in the survey.**

Estimates of ***e*** (proportion of non-respondents with unknown eligibility presumed to be eligible) based on non-response audits of random samples of non-respondents.

Municipality/city (Audit size = 150)

0 Confirmed ineligible 6.7%

1 Confirmed eligible 87.3

2 Unable to confirm 6.0

Allocate half of uncertain to eligible 🡪 ***e*** = 90.3%

County (Audit size = 150)

0 Confirmed ineligible 5.3%

1 Confirmed eligible 89.3

2 Unable to confirm 5.3

Allocate half of uncertain to eligible 🡪 ***e*** = 92.0%

Superintendents (

0 Confirmed ineligible 6.7%

1 Confirmed eligible 82.7

2 Unable to confirm 10.7

Allocate half of uncertain to eligible 🡪 ***e*** = 88.0%

**C.1.3. AAPOR Response Rate RR4:**

To calculate response rates, we employ the AAPOR [2] RR4 formula that accounts for the proportion of the recruitment sample that is eligible to complete the survey :

$$RR4= \frac{Completes+Usable Partial}{\left( Completes+Usable Partial \right)+\left( Refusals \right)+e(Unknown)}$$

Based on the dispositions reported in Appendix section C.1.2, we derive the following response rates:

*Calculations for municipal and county officials*

$$RR4= \frac{1,096+127}{\left( 1,096+127 \right)+\left( 228 \right)+0.90(12,094)}=9.9\%$$

*Calculations for superintendent sample*

$$RR4= \frac{102+15}{\left( 102+15 \right)+\left( 17 \right)+0.88(3,607)}=3.5\%$$

*Calculations for full sample*

$$RR4= \frac{1,198+142}{\left( 1,198+142 \right)+\left( 245 \right)+0.90(15,701)}=8.5\%$$

**C.2. Correlates of non-response**

To explore potential patterns in non-response that could make the resulting analysis dataset less representative than the full recruitment sample, we estimated a logistic regression model where the dependent variable is coded 1 if a recruited official responded and 0 otherwise.

Because the response rate was so much lower for the sample of superintendents, we conduct the analysis first for city and county officials, and then separately for school superintendents.

Of course, we know little about non-respondents. But we do know quite a bit about their locale and we derived measures of their social and political environment based on available data. Continuous measures were binned into quartiles with the lowest quartile serving as the omitted (contrast) category in the model.

The model includes the following independent variables:

City (=1) or county (0)

Official was contacted by email in addition to postal letter (1) or not contacted by email (0)

Jurisdiction population size

Proportion of adult (25+) jurisdiction population holding a college degree

Proportion of county population estimated to be an adherent of Evangelical religious body

Proportion of county population estimated to be an adherent of Catholic religious body

Republican share of the 2020 presidential county vote

Proportion of county population estimated to be African American

Proportion of county population estimated to be Hispanic

**C.2.1. Model results for city and county officials**

Table C1, below shows that the most important predictor is whether we began the survey with an email address on file for the official. Those contacted only through post had much lower odds of responding.

We also see higher response rates from officials in locales with more educated adult populations and slightly lower response rates in areas that were the most Catholic, the most African American. Despite the large sample size, local presidential vote was not significantly related to non-response.

| **Table C1. Logistic regression model of survey response (N = 13,428 city and county officials)** | | |  |
| --- | --- | --- | --- |
|  |  |  |  |
|  | B | Std Error |  |
|  |  |  |  |
| Municipality (versus county) | -0.045 | (0.065) |  |
| Has email | 0.746 | (0.254) | * |
| Population quartile |  |  |  |
| Small | 0.271 | (0.103) | * |
| Medium | 0.063 | (0.116) |  |
| Large | -0.093 | (0.129) |  |
| Adults 25+ with college degree quartile |  |  |  |
| 2nd quartile | 0.197 | (0.102) |  |
| 3rd quartile | 0.394 | (0.104) | * |
| Top quartile | 0.452 | (0.113) | * |
| Evangelical adherents quartile |  |  |  |
| 2nd quartile | 0.178 | (0.087) | * |
| 3rd quartile | 0.012 | (0.095) |  |
| Top quartile | -0.031 | (0.115) |  |
| Catholic adherents quartile |  |  |  |
| 2nd quartile | 0.067 | (0.096) |  |
| 3rd quartile | -0.054 | (0.105) |  |
| Top quartile | -0.234 | (0.116) | * |
| Republican share of 2020 presidential vote |  |  |  |
| 2nd quartile | 0.010 | (0.088) |  |
| 3rd quartile | 0.116 | (0.098) |  |
| Top quartile | -0.031 | (0.125) |  |
| African American share of population |  |  |  |
| 2nd quartile | -0.101 | (0.091) |  |
| 3rd quartile | -0.157 | (0.096) |  |
| Top quartile | -0.297 | (0.106) | * |
| Hispanic share of population |  |  |  |
| 2nd quartile | -0.034 | (0.092) |  |
| 3rd quartile | 0.049 | (0.092) |  |
| Top quartile | 0.044 | (0.106) |  |
|  |  |  |  |
| Constant | -3.249 | (0.296) | * |
|  |  |  |  |
| * p<0.05 |  |  |  |

To place the logit estimates in context, see Table C2, which provides the estimated RR4 response rate by respondent and community characteristics along with the Wald test from the logistic regression which tests the null hypothesis that the set of dummy indicators all have an effect equal to zero.

The table shows the dramatic impact of being able to contact officials by email (10%) versus relying on only postal recruitment (5%). Notably, however, this is the only substantively large effect. In every other instance, response rates within levels of a variable vary in a narrow range of 8%-12% suggesting that we achieved the intended broad representativeness we desired.

| **Table C2. Response rates by sample and community characteristics (N = 13,428 city and county officials).** | | | | |
| --- | --- | --- | --- | --- |
|  |  |  |  |  |
|  | Response rate |  |  | Response rate |
| **Type of jurisdiction** |  |  | **Has email** |  |
| County (43%) | 0.10 |  | No (2%) | 0.05 |
| Municipality (56%) | 0.10 |  | Yes (97%) | 0.10 |
| *Wald test: p = 0.44 (ns)* |  |  | *Wald test: p = 0.00* |  |
| **Community size** |  |  | **Adults with college degree** |  |
| Very small (18%) | 0.09 |  | Lowest quartile (22%) | 0.08 |
| Small (23%) | 0.12 |  | 2nd quartile (24%) | 0.10 |
| Medium (26%) | 0.10 |  | 3rd quartile (27%) | 0.11 |
| Large (31%) | 0.09 |  | Top quartile (25%) | 0.11 |
| *Wald test: p = 0.00* |  |  | *Wald test: p = 0.00* |  |
| **Evangelical adherents** |  |  | **Catholic adherents** |  |
| Lowest quartile (25%) | 0.10 |  | Lowest quartile (23%) | 0.09 |
| 2nd quartile (24%) | 0.11 |  | 2nd quartile (25%) | 0.11 |
| 3rd quartile (26%) | 0.10 |  | 3rd quartile (26%) | 0.10 |
| Top quartile (23%) | 0.09 |  | Top quartile (24%) | 0.09 |
| *Wald test: p = 0.02* |  |  | *Wald test: p = 0.02* |  |
| **Black Pct** |  |  | **Hispanic Pct** |  |
| Lowest quartile (21%) | 0.11 |  | Lowest quartile (22%) | 0.10 |
| 2nd quartile (24%) | 0.11 |  | 2nd quartile (25%) | 0.10 |
| 3rd quartile (26%) | 0.10 |  | 3rd quartile (26%) | 0.11 |
| Top quartile (27%) | 0.08 |  | Top quartile (25%) | 0.09 |
| *Wald test: p = 0.05* |  |  | *Wald test: p = 0.76 (ns)* |  |
| **Republican share of vote** |  |  |  |  |
| Lowest quartile (26%) | 0.10 |  |  |  |
| 2nd quartile (25%) | 0.10 |  |  |  |
| 3rd quartile (25%) | 0.11 |  |  |  |
| Top quartile (22%) | 0.09 |  |  |  |
| *Wald test: p = 0.22 (ns)* |  |  |  |  |

**Appendix D. Analysis Weights**

**D.1. Base weights**

The sampling design described in Appendix A is intended to generalize our results to the population of constituents, rather than the population of all elected officials – this is because the latter strategy would be dominated by officials who represent less than 5% of the US population.

Consequently, the base weights (or *design weights*) are all equal to 1.00. In other words, our recruitment sample is designed to represent officials in proportion to the number of constituents they serve.

**D.1.1 Technical note on base weights**

In theory, users who wish to generalize to the population of all officials, could calculate an alternative base weight that is the inverse of the probability of selection. However, we do not recommend doing so as this would mean that the 56 sampled officials from tiny jurisdictions (e.g., cities with fewer than 2000 residents) would contribute more than half the statistical information used in any analysis. That is, these 56 officials would dominate models and statistics calculated from the full sample of 1000+ completed interviews.

**D.2. Creating analysis weight for differential non-response**

Of course, the final analysis dataset is not a random sample of the recruitment file, due to differential non-response as described in Appendix C. An individual’s latent propensity to respond will reflect whether we had an email address for them, whether or not they lived in an area of high educational attainment, with large or small numbers of racial and ethnic minorities and so on. If one responded had latent response probability of 0.15, then they are speaking on behalf of about 6 other sampled officials with similar characteristics who did not respond. In contrast, someone with a response propensity of 0.10 is speaking on behalf of 9 sampled officials from similar locales who did not respond. To balance the analysis data set, inverse propensity weights boost the voice of those representing more non-respondents. This non-response adjustment weight is calculated as follows:

$${AnalysisWeight}_{i}={Baseweight}_{i} \times\frac{1}{{ResponsePropensity}_{i}}$$

Where *ResponsePropensity****_i_*** is the predicted propensity to respond of respondent ***i*** based on their community’s characteristics as modeled in Table C.1. The distribution of these propensities is show in Figure D1.

Figure D1. Response propensities for city and county officials.

Following standard practice to minimizes the number of influential outliers [3-4] these propensities were binned into deciles, with the decile mean used in these calculations.

The resulting analysis weight has a mean of 1.00, a standard deviation of 0.27, and ranges from 0.659 to 1.757. The low ratio of the standard deviation to the mean suggests that the weights will not appreciably impact analyses (weighted and unweighted analyses will be similar) and have a minimal design effect.

**Appendix E. Exact Question Wording**

**E.1. Dependent Variables**

Thinking about last year’s mid-term elections in November, how confident are you that votes in your **county or city** were counted as voters intended?”

Not at all confident

Not too confident

Somewhat confident

Very confident

“Now think about vote counting throughout the **country**. How confident are you that votes nationwide were counted as voters intended?”

Not at all confident

Not too confident

Somewhat confident

Very confident

**E.2. Key Independent Variables**

***Partisan identification***

Generally speaking, do you think of yourself as a…?

Democrat

[If ticked] Would you call yourself a strong Democrat or a not very strong Democrat?

Republican

[If ticked] Would you call yourself a strong Republican or a not very strong Republican?

Independent

[If ticked] Do you think of yourself as closer to the Democratic or the Republican Party?

Other

[If ticked] Do you think of yourself as closer to the Democratic or the Republican Party?

Not sure

[If ticked] Do you think of yourself as closer to the Democratic or the Republican Party?

***Trust***

How much do you trust each of the following people to always do the right thing?

Your family

People who live in your neighborhood

People you meet for the first time

People from a different religion

People from a different political party

People managing the agencies in your state government

Completely,

Somewhat

Not very much

Not at all

***Political Ambition***

How would you characterize your interest in holding higher elected office in the future?

I have no interest in holding higher elected office at any time in the future [0]

I am open to the possibility of holding higher elected office in the future [coded 1]

I am actively considering running for higher elected office [coded 1]

**E.2. Control Variables**

Age [What year were born?]

Educ [What is the highest level of school you have completed?]

Less than high school

High school

Some college or trade school

Associate’s degree

Bachelor’s degree

Graduate or Professional degree

Doctoral Degree

Recoded to two binary measures: holds college degree, holds graduate degree

Race/Eth, [Which of the following describes you? (Please check all that apply)]

White (1)

Black or African American (2)

Hispanic or Latino (3)

Asian or Asian American (4)

Native Hawaiian or Pacific Islander (5)

American Indian or Native American (6)

Middle Eastern or North African (7)

Some other race or ethnicity (specify) (8)

Gender identity: Are you a:

Man

Woman,

Something else

Sexuality: Which of the following best represents how you think of yourself?

Lesbian or gay

Heterosexual or straight

Bisexual

Prefer not to answer

Don’t know

[Answers to these two questions were utilized to create the following variables:

Female (1) versus all else

LGBQT (=1 if any of the following are selected:

Lesbian or gay

Bisexual

“Something else” on gender identity and not heterosexual on sexual preference question.

Married

Which of the following best describes your marital or relationship status?

Divorced (0)

Living with a partner (1)

Married or civil union (1)

Separated (0)

Never married (0)

Widow/Widower (0)

Prefer not to say (0)

Children 18 or under: How many children age 0 to 17 live in your household?

[recoded to 0, 1, 2, 3, 4 or more]

Religious affiliation (RELTRAD): Answers to the following questions were used sort cases into religious tradition categories:

What is your present religion, if any? Protestant (for example, Baptist, Methodist, Non-denominational, Lutheran, Presbyterian, Pentecostal, Episcopalian, Reformed, Church of Christ, Adventists, etc.), Roman Catholic (Catholic), Orthodox (such as Greek, Russian, or some other Orthodox church), Mormon (Church of Jesus Christ of Latter-Day Saints or LDS), Just Christian, Unitarian, Jewish, Muslim, Buddhist, Hindu, Atheist, Agnostic, Nothing in particular, Something else (please specify)

IF Nothing in particular:

Would you say you are: Atheist, Agnostic, Christian, or Nothing in particular?

IF ANY OF THE ABOVE, EXCEPT: Unitarian, Jewish, Muslim, Buddhist, Hindu, Atheist:

Do you consider yourself as a born-again or evangelical Christian?

IF ANY OF THE ABOVE, EXCEPT: Unitarian, Jewish, Muslim, Buddhist, Hindu, Atheist:

Which of these comes closest to describing your view: The Bible is the actual word of God and is to be taken literally, word for word; The Bible is the inspired word of God by not everything in it should be taken literally, word for word; The Bible is a book written by humans and is not the word of God; I don’t have an opinion about this; Other

RELTRAD (religious traditions classification):

Conservative Protestant: Protestant/Just Christian/Christian and Not Black and born again or Bible literalist

Mainline Protestant: Protestant/Just Christian/Christian and Not Black and not born again and not Bible literalist

Black Protestant: Protestant/Just Christian/Christian and Black

Catholic

Jewish

Other: Orthodox, LDS, Unitarian, Muslim, Buddhist, Hindu, or Something Else

None: Atheist, Agnostic, or Nothing in Particular

Religiosity: Standardized scale (alpha=.87) comprised of:

How often do you pray, other than at meals or worship services?

Never

A few times a year

Monthly

Weekly

Daily

Aside from weddings and funerals, how often do you attend religious services?

Never

A few times a year

Once or twice a month

Almost every week

Every week

To what extent do you consider yourself a spiritual person? Are you…

Not spiritual at all

Slightly spiritual

Moderately spiritual

Very spiritual

Would you say that religion provides … in your day to day life?

No guidance

Some guidance

Quite a bit of guidance

A great deal of guidance

Seniority

How many years and months have you been in your current local government position? Please enter numbers for both fields.

[Recoded to

Under 2 years

2-5 years

5-10 years

10 or more years

Member of military household

Have you or someone in your household ever served in the U.S. military, military reserves, or National Guard?

Yes, myself (1)

Yes, a household member (1)

Yes, myself and a household member (1)

No (0)

**Appendix F. Non-survey Constructed Variables**

**F1. Key Independent variables**

***Local Responsibility for Election Administration***

This variable is coded 1 when an official serves a jurisdiction (county or municipality) that has primary responsibility for election administration.

In 37 states, all county officials are coded 1 and all municipal officials are coded 0. In six New England States, all municipal officials are coded 1 with county officials coded 0. Alaska is coded 0 for all officials as they use large election districts that each encompass multiple counties and municipalities. For four states with mixed responsibilities (HI, IL, MO, VA) most county officials are coded 1, but that is reversed for specific cities with special carve outs. Finally, Michigan and Wisconsin have complicated systems that provide “joint custody,” with shared responsibility for election administration (e.g., counties approve ballots and conduct post-election canvas and audits, while cities maintain voter roles and are responsible for polling places, technology and election day staffing). In these states, all officials are deemed living in a locale with election jurisdiction and coded 1.

***County Partisan Context***

Republican share of the 2020 presidential two-party vote (this is recoded into increments of 10 percentage points (e.g., 53% of the county vote is coded as 5.3; in this way, a one-unit change in X represents a ten-percentage-point increase a vote share).

***State Leadership Election Denial Score***

We utilize data compiled by journalists at FiveThirtyEight in the Fall of 2022.

For each statewide candidate we created an ordinal scale according to the following rules.

Table F1.

| FiveThirtyEight classification | Our denial score |
| --- | --- |
| Fully denied legitimacy of Biden election | 3 |
| Accepted Biden election, but with reservations | 2 |
| Neither accept nor deny, but raised questions | 2 |
| Avoided answering | 1 |
| No comment | 1 |
| Full accepted Biden election | 0 |
|  |  |

We then calculated the mean score across all candidates in each state.

**F2. Control variables**

***From county sample***

This is a dummy variable coded 1 if the official’s jurisdiction is a county and 0 if it is a municipality.

***Community size***

Community size (quintile)

**Appendix G. Supplementary Tables**

| Table G.1. Descriptive statistics | |  |  |  |  |  |  |  |
| --- | --- | --- | --- | --- | --- | --- | --- | --- |
|  |  |  |  |  |  |  |  |  |
| ***A. Numeric variables*** |  |  |  |  |  |  |  |  |
|  |  |  |  |  | Percentiles | | |  |
| Variable | n | Mean | S.D. |  | 25th | 50th | 75th |  |
| Party identification (0-6) | 687 | 2.8 | 2.5 |  | 0.0 | 3.0 | 5.0 |  |
| Trump % of 2020 vote ÷ 10 (1-9) | 787 | 4.9 | 1.6 |  | 3.8 | 4.8 | 6.0 |  |
| Social trust scale (-3.9 -2.9) | 743 | 0.0 | 1.0 |  | -0.7 | -0.2 | 0.6 |  |
| Age (24 - 90) | 669 | 59.8 | 12.6 |  | 51.0 | 61.0 | 69.0 |  |
| Religiosity index (-1.6 - 1.4) | 696 | 0.1 | 0.82 |  | -0.58 | 0.12 | 0.82 |  |
|  |  |  |  |  |  |  |  |  |
|  |  |  |  |  |  |  |  |  |
| ***B. Binary variables*** |  |  |  |  |  |  |  |  |
|  |  |  |  |  |  |  |  |  |
|  | n | Proportion |  |  |  | n | Proportion |  |
| Jurisdiction administers elections | 787 | 0.38 |  | Hispanic |  | 682 | 0.06 |  |
| Ambitious for higher office | 787 | 0.53 |  | Black |  | 682 | 0.08 |  |
| Military household | 694 | 0.37 |  | Female |  | 787 | 0.29 |  |
| Is married | 787 | 0.68 |  | LGBQT |  | 787 | 0.05 |  |
|  |  |  |  |  |  |  |  |  |
|  |  |  |  |  |  |  |  |  |
| ***C. Ordinal and categorical variables*** | |  |  |  |  |  |  |  |
|  |  |  |  |  |  |  |  |  |
| Children under 18 in HH | Pctg |  | Seniority in current position | | | Pctg |  |  |
| None | 76.4 |  | Under 2 yrs | |  | 21.9 |  |  |
| One | 8.6 |  | 2-5 yrs |  |  | 31.7 |  |  |
| Two | 9.3 |  | 5-10 yrs |  |  | 23.5 |  |  |
| Three or more | 5.7 |  | 10 or more yrs | |  | 22.9 |  |  |
| Total | 100.0 |  | Total |  |  | 100.0 |  |  |
|  |  |  |  |  |  |  |  |  |
| Highest degree earned | Pctg |  | Religious tradition | |  | Pctg |  |  |
| Less than Bachelor | 22.1 |  | Conservative Protestant | | | 22.0 |  |  |
| College degree | 32.6 |  | Mainline Protestant | |  | 25.5 |  |  |
| Graduate degree | 45.3 |  | Black Protestant | |  | 6.3 |  |  |
| Total | 100.0 |  | Catholic |  |  | 21.8 |  |  |
|  |  |  | Jewish |  |  | 3.2 |  |  |
| Community population quintile | Pctg |  | Other |  |  | 6.4 |  |  |
| First | 23.6 |  | None |  |  | 14.9 |  |  |
| Second | 17.0 |  | Total |  |  | 100.0 |  |  |
| Third | 19.8 |  |  |  |  |  |  |  |
| Fourth | 20.6 |  |  |  |  |  |  |  |
| Fifth | 19.0 |  |  |  |  |  |  |  |
| Total | 100.0 |  |  |  |  |  |  |  |
|  |  |  |  |  |  |  |  |  |

|  |  |  |  |  |  |  |  |
| --- | --- | --- | --- | --- | --- | --- | --- |

Table G.2 Full models used to produce figure 2 (logit estimates with standard errors in parentheses).

| Table G2: Logit Models Predicting Confidence in Local and National Elections | | | | |
| --- | --- | --- | --- | --- |
|  |  |  |  |  |
|  | Local confidence | | National confidence | |
| Confidence in county/city elections | |  | 1.987 | ** |
|  |  |  | (0.207) |  |
| In local responsible for elections | 0.498 |  | 0.188 |  |
|  | (0.270) |  | (0.204) |  |
| Party ID (0-6) | -0.303 | ** | -0.433 | ** |
|  | (0.074) |  | (0.055) |  |
| 2020 Trump vote (0-10) | 0.213 |  | -0.127 |  |
|  | (0.133) |  | (0.080) |  |
| Social trust (z-score) | 0.368 | * | 0.213 | * |
|  | (0.158) |  | (0.107) |  |
| Open to higher office in future | -0.751 | * | -0.376 |  |
|  | (0.330) |  | (0.211) |  |
| Mean GOP candidate denial score | -0.472 |  | 0.482 | * |
|  | (0.312) |  | (0.220) |  |
| Community size (1-5) | 0.090 |  | 0.054 |  |
|  | (0.133) |  | (0.081) |  |
| Years in current position | 0.073 |  | 0.073 |  |
|  | (0.144) |  | (0.107) |  |
| Official or HH member served in military | -0.368 |  | -0.093 |  |
|  | (0.304) |  | (0.221) |  |
| Education |  |  |  |  |
| College degree | 0.723 | * | 0.477 |  |
|  | (0.346) |  | (0.277) |  |
| Graduate degree | 1.223 | ** | 0.652 | * |
|  | (0.331) |  | (0.272) |  |
| Hispanic/Latino Race/Ethnicity | 0.238 |  | 0.372 |  |
|  | (0.551) |  | (0.561) |  |
| Married or civil union | 0.169 |  | 0.404 |  |
|  | (0.373) |  | (0.265) |  |
| Children age 0 to 17 live in household | |  |  |  |
| One | 0.827 |  | 0.009 |  |
|  | (0.533) |  | (0.439) |  |
| Two | -0.287 |  | 0.022 |  |
|  | (0.550) |  | (0.417) |  |
| Three or more | -0.001 |  | -0.192 |  |
|  | (0.569) |  | (0.419) |  |
| Female | -0.082 |  | 0.334 |  |
|  | (0.318) |  | (0.238) |  |
| Identifies as LGBQ or T | 0.867 |  | 0.208 |  |
|  | (1.167) |  | (0.577) |  |
| Age in years | -0.025 |  | -0.001 |  |
|  | (0.018) |  | (0.012) |  |
| Religious tradition (7 cats) |  |  |  |  |
| Mainline Protestant | 0.973 | * | 0.886 | ** |
|  | (0.411) |  | (0.318) |  |
| Black Protestant | 1.296 |  | 0.304 |  |
|  | (0.869) |  | (0.584) |  |
| Catholic | 0.073 |  | 0.369 |  |
|  | (0.355) |  | (0.310) |  |
| Jewish | 15.503 | ** | 1.163 |  |
|  | (0.565) |  | (0.744) |  |
| Other | 1.296 |  | 1.305 | ** |
|  | (0.689) |  | (0.416) |  |
| None | 1.350 | * | 1.341 | ** |
|  | (0.575) |  | (0.473) |  |
| Religiosity | 0.131 |  | 0.229 |  |
|  | (0.219) |  | (0.172) |  |
| cut1 | -5.433 |  | 4.718 |  |
|  | (1.668) |  | (1.282) |  |
| cut2 | -4.195 |  | 6.015 |  |
|  | (1.630) |  | (1.280) |  |
| cut3 | -2.419 |  | 8.306 |  |
|  | (1.611) |  | (1.323) |  |
|  |  |  |  |  |
|  |  |  |  |  |
| Observations | 570 |  | 567 |  |
| ** p<.01, * p<.05 |  |  |  |  |
|  |  |  |  |  |

Table G.3 Full models used to produce figure 3 (logit estimates with standard errors in parentheses).

| Table G3: Logit Models Predicting Confidence in Local and National Elections (GOP officials only) | | | | | |
| --- | --- | --- | --- | --- | --- |
|  |  |  |  |  |  |
|  | Local confidence | | National confidence | |  |
| Confidence in county/city elections |  |  | 1.716 | ** |  |
|  |  |  | (0.241) |  |  |
| In local responsible for elections | 0.494 |  | 0.045 |  |  |
|  | (0.320) |  | (0.272) |  |  |
| Party ID (0-6) | -0.018 |  | -0.597 | ** |  |
|  | (0.238) |  | (0.225) |  |  |
| 2020 Trump vote (0-10) | 0.328 | * | -0.093 |  |  |
|  | (0.160) |  | (0.115) |  |  |
| Social trust (z-score) | 0.433 | * | 0.236 |  |  |
|  | (0.192) |  | (0.130) |  |  |
| Open to higher office in future | -0.259 |  | 0.207 |  |  |
|  | (0.366) |  | (0.284) |  |  |
| Mean GOP candidate denial score | -0.819 | * | -0.452 |  |  |
|  | (0.385) |  | (0.295) |  |  |
| Community size (1-5) | 0.025 |  | 0.162 |  |  |
|  | (0.173) |  | (0.129) |  |  |
| Years in current position | 0.086 |  | 0.153 |  |  |
|  | (0.180) |  | (0.138) |  |  |
| Official or HH member served in military | -0.226 |  | 0.023 |  |  |
|  | (0.339) |  | (0.275) |  |  |
| Education |  |  |  |  |  |
| College degree | 0.605 |  | 0.465 |  |  |
|  | (0.424) |  | (0.369) |  |  |
| Graduate degree | 1.079 | ** | 0.770 | * |  |
|  | (0.404) |  | (0.345) |  |  |
| Hispanic/Latino Race/Ethnicity | -0.049 |  | 0.293 |  |  |
|  | (0.617) |  | (1.048) |  |  |
| Married or civil union | -0.341 |  | 0.240 |  |  |
|  | (0.552) |  | (0.422) |  |  |
| Children age 0 to 17 live in household |  |  |  |  |  |
| One | 0.401 |  | 0.080 |  |  |
|  | (0.613) |  | (0.603) |  |  |
| Two | -0.683 |  | -0.540 |  |  |
|  | (0.697) |  | (0.674) |  |  |
| Three or more | -0.368 |  | -0.242 |  |  |
|  | (0.635) |  | (0.534) |  |  |
| Female | -0.222 |  | 0.216 |  |  |
|  | (0.425) |  | (0.363) |  |  |
| Identifies as LGBQ or T | 14.058 | ** | 0.004 |  |  |
|  | (1.097) |  | (1.502) |  |  |
| Age in years | -0.048 | * | -0.016 |  |  |
|  | (0.022) |  | (0.017) |  |  |
| Religious tradition (7 cats) |  |  |  |  |  |
| Mainline Protestant | 1.154 | * | 0.709 |  |  |
|  | (0.523) |  | (0.424) |  |  |
| Black Protestant | 0.515 |  | 1.509 | ** |  |
|  | (1.418) |  | (0.544) |  |  |
| Catholic | 0.716 |  | 0.523 |  |  |
|  | (0.444) |  | (0.400) |  |  |
| Jewish | 16.166 | ** | 0.790 |  |  |
|  | (1.320) |  | (0.984) |  |  |
| Other | 0.719 |  | 1.124 | * |  |
|  | (0.799) |  | (0.511) |  |  |
| None | 1.521 | * | 0.997 |  |  |
|  | (0.724) |  | (0.814) |  |  |
| Religiosity | 0.423 |  | 0.209 |  |  |
|  | (0.271) |  | (0.259) |  |  |
| cut1 | -4.658 |  | 1.955 |  |  |
|  | (2.357) |  | (1.946) |  |  |
| cut2 | -3.544 |  | 3.150 |  |  |
|  | (2.354) |  | (1.938) |  |  |
| cut3 | -1.705 |  | 5.524 |  |  |
|  | (2.351) |  | (1.942) |  |  |
|  |  |  |  |  |  |
|  |  |  |  |  |  |
| Observations | 256 |  | 255 |  |  |
| ** p<.01, * p<.05 |  |  |  |  |  |
|  |  |  |  |  |  |

Table G.4 Models used to test ambition  **×** denialism interaction (logit estimates with standard errors in parentheses).

| Table G4: Logit Models Predicting Confidence in Local and National Elections, with ambition x denialism interaction (GOP officials only) | | | | |
| --- | --- | --- | --- | --- |
|  | Local confidence | | National confidence | |
| Confidence that county/city elections |  |  | 1.719 | ** |
|  |  |  | (0.243) |  |
| In locale responsible for elections | 0.495 |  | 0.044 |  |
|  | (0.320) |  | (0.272) |  |
| Party ID (0-6) | -0.019 |  | -0.590 | * |
|  | (0.236) |  | (0.227) |  |
| 2020 Trump vote (0-10) | 0.327 | * | -0.091 |  |
|  | (0.160) |  | (0.114) |  |
| Social trust (z-score) | 0.433 | * | 0.236 |  |
|  | (0.193) |  | (0.131) |  |
|  |  |  |  |  |
| Open to higher office in future | -0.715 |  | -1.012 |  |
|  | (1.318) |  | (1.076) |  |
| Mean GOP candidate denial score | -0.228 |  | 0.048 |  |
|  | (0.569) |  | (0.445) |  |
|  |  |  |  |  |
| Ambition x Denial Score | -0.054 |  | 0.296 |  |
|  | (0.682) |  | (0.572) |  |
| Community size (1-5) | 0.024 |  | 0.166 |  |
|  | (0.173) |  | (0.129) |  |
| Years in current position | 0.086 |  | 0.150 |  |
|  | (0.182) |  | (0.138) |  |
| Official or HH member served in military | -0.228 |  | 0.046 |  |
|  | (0.340) |  | (0.285) |  |
| Education |  |  |  |  |
| College degree | 0.606 |  | 0.457 |  |
|  | (0.423) |  | (0.371) |  |
| Graduate degree | 1.081 | ** | 0.773 | * |
|  | (0.404) |  | (0.347) |  |
| Hispanic/Latino Race/Ethnicity | -0.043 |  | 0.258 |  |
|  | (0.615) |  | (1.047) |  |
| Married or civil union | -0.342 |  | 0.242 |  |
|  | (0.552) |  | (0.421) |  |
| Children age 0 to 17 live in household |  |  |  |  |
| One | 0.403 |  | 0.067 |  |
|  | (0.618) |  | (0.605) |  |
| Two | -0.683 |  | -0.556 |  |
|  | (0.698) |  | (0.677) |  |
| Three or more | -0.363 |  | -0.271 |  |
|  | (0.642) |  | (0.542) |  |
| Female | -0.221 |  | 0.213 |  |
|  | (0.426) |  | (0.363) |  |
| Identifies as LGBQ or T | 14.058 | ** | -0.017 |  |
|  | (1.095) |  | (1.484) |  |
| Age in years | -0.048 | * | -0.016 |  |
|  | (0.022) |  | (0.018) |  |
| Religious tradition (7 cats) |  |  |  |  |
| Mainline Protestant | 1.157 | * | 0.689 |  |
|  | (0.527) |  | (0.425) |  |
| Black Protestant | 0.509 |  | 1.535 | ** |
|  | (1.413) |  | (0.541) |  |
| Catholic | 0.714 |  | 0.530 |  |
|  | (0.443) |  | (0.403) |  |
| Jewish | 16.169 | ** | 0.779 |  |
|  | (1.330) |  | (1.003) |  |
| Other | 0.715 |  | 1.142 | * |
|  | (0.803) |  | (0.516) |  |
| None | 1.517 | * | 1.019 |  |
|  | (0.720) |  | (0.811) |  |
| Religiosity | 0.423 |  | 0.199 |  |
|  | (0.272) |  | (0.263) |  |
|  |  |  |  |  |
|  |  |  |  |  |
| cut1 | -4.608 |  | 1.702 |  |
|  | (2.505) |  | (2.052) |  |
| cut2 | -3.494 |  | 2.898 |  |
|  | (2.511) |  | (2.038) |  |
| cut3 | -1.655 |  | 5.276 |  |
|  | (2.517) |  | (2.043) |  |
|  |  |  |  |  |
| Observations | 256 |  | 255 |  |
| ** p<.01, * p<.05 |  |  |  |  |

**References cited in the supporting information:**

1. U.S. Census Bureau. Governments master address file, 2021. Available from: https://www.census.gov/data/datasets.html
2. American Association for Public Opinion Research. Standard definitions: final dispositions of case codes and outcome rates for surveys*.* 10th edition. 2023. Available from: https://aapor.org/standards-and-ethics/standard-definitions/
3. Bethlehem, JG, Cobben F, Schouten B. Handbook of nonresponse in household surveys. Hoboken, NJ: John Wiley & Sons; 2011.
4. Valliant, R, Dever JA. Survey weights: a step-by-step guide to calculation. College Station, TX: Stata Press; 2018.
